# Pediatric T-ALL type-1 and type-2 relapses develop along distinct pathways of clonal evolution

**DOI:** 10.1038/s41375-022-01587-0

**Published:** 2022-05-18

**Authors:** Paulina Richter-Pechańska, Joachim B. Kunz, Tobias Rausch, Büşra Erarslan-Uysal, Beat Bornhauser, Viktoras Frismantas, Yassen Assenov, Martin Zimmermann, Margit Happich, Caroline von Knebel-Doeberitz, Nils von Neuhoff, Rolf Köhler, Martin Stanulla, Martin Schrappe, Gunnar Cario, Gabriele Escherich, Renate Kirschner-Schwabe, Cornelia Eckert, Smadar Avigad, Stefan M. Pfister, Martina U. Muckenthaler, Jean-Pierre Bourquin, Jan O. Korbel, Andreas E. Kulozik

**Affiliations:** 1 Department of Pediatric Oncology, Hematology, and Immunology, University of Heidelberg, Heidelberg, Germany; 2 Hopp Children´s Cancer Center Heidelberg (KiTZ), Heidelberg, Germany; 3 Molecular Medicine Partnership Unit (MMPU), EMBL and Medical Faculty of the University of Heidelberg, Heidelberg, Germany; 4 European Molecular Biology Laboratory (EMBL), Heidelberg, Germany; 5 Division of Pediatric Oncology, University Children’s Hospital, Zürich, Switzerland; 6 Division of Pediatric Neurooncology, German Cancer Consortium (DKTK) and German Cancer Research Center (DKFZ), Heidelberg, Germany; 7 Department of Pediatric Hematology and Oncology, Hannover Medical School, Hannover, Germany; 8 Department of Pediatrics III, University Hospital, University of Duisburg-Essen, Essen, Germany; 9 Institute of Human Genetics, University of Heidelberg, Heidelberg, Germany; 10 Department of Pediatrics, University Hospital Schleswig-Holstein, Campus Kiel, Kiel, Germany; 11 Clinic of Pediatric Hematology and Oncology, University Medical Center Hamburg-Eppendorf, Hamburg, Germany; 12 Department of Pediatric Oncology/Hematology, Charité Universitätsmedizin Berlin, Berlin, Germany; 13 German Cancer Consortium (DKTK), and German Cancer Research Center (DKFZ), Heidelberg, Germany; 14 Molecular Oncology, Felsenstein Medical Research Center and Pediatric Hematology Oncology, Schneider Children’s Medical Center of Israel, Petah Tikva, Israel

**Keywords:** Cancer genomics, Cancer genomics, Leukaemia

## Abstract

The mechanisms underlying T-ALL relapse remain essentially unknown. Multilevel-omics in 38 matched pairs of initial and relapsed T-ALL revealed 18 (47%) type-1 (defined by being derived from the major ancestral clone) and 20 (53%) type-2 relapses (derived from a minor ancestral clone). In both types of relapse, we observed known and novel drivers of multidrug resistance including *MDR1* and *MVP*, *NT5C2* and JAK-STAT activators. Patients with type-1 relapses were specifically characterized by *IL7R* upregulation. In remarkable contrast, type-2 relapses demonstrated (1) enrichment of constitutional cancer predisposition gene mutations, (2) divergent genetic and epigenetic remodeling, and (3) enrichment of somatic hypermutator phenotypes, related to *BLM, BUB1B*/*PMS2 and TP53* mutations. T-ALLs that later progressed to type-2 relapses exhibited a complex subclonal architecture, unexpectedly, already at the time of initial diagnosis. Deconvolution analysis of ATAC-Seq profiles showed that T-ALLs later developing into type-1 relapses resembled a predominant immature thymic T-cell population, whereas T-ALLs developing into type-2 relapses resembled a mixture of normal T-cell precursors. In sum, our analyses revealed fundamentally different mechanisms driving either type-1 or type-2 T-ALL relapse and indicate that differential capacities of disease evolution are already inherent to the molecular setup of the initial leukemia.

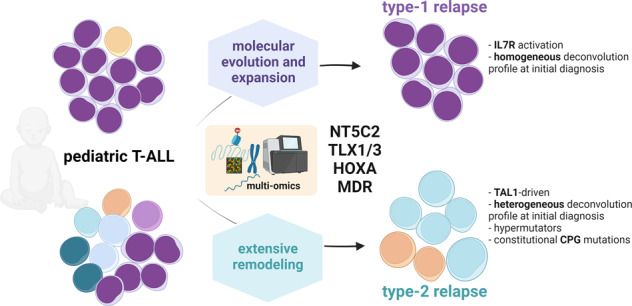

## Introduction

Relapse is the main cause of death from pediatric acute precursor T-cell leukemia (T-ALL) [1, 2], but the underlying mechanisms of disease evolution from initial disease to relapse remain incompletely understood and show a remarkable interpatient heterogeneity. The acquisition of mutations and epigenetic modifications represent important components of this process [3–7]. Specifically, activating mutations of the nucleosidase NT5C2 are acquired by app. 20% of patients at relapse [8–10]. These mutations are predicted to confer chemotherapy resistance but are often found to be subclonal [10]. *TP53* mutations occur in-app. 12% of relapses and predict a high risk of treatment failure and fatal outcome [11–13]. Although prevalent at T-ALL relapse [14] and correlated with an increased cumulative incidence of relapse [15], RAS-MAPK pathway-activating mutations were reported both, in clones that are eradicated and in those that emerge at relapse at the same time rendering lymphoblasts resistant against methotrexate, while sensitizing them to vincristine [4]. Similarly, *NOTCH1* activating mutations, which enhance proliferation and survival [16] have recently been reported to be frequently acquired at later T-ALL stages thus highlighting the importance of *NOTCH1*-activation for T-ALL progression [17].

We have previously described two types of T-ALL relapses, characterized either by the clonal evolution of the major clone present at the time of initial presentation (type-1) or by the emergence and evolution of a minor ancestor clone showing a molecular profile that is distinct from the predominant initial clone (type-2) [3].

As cross-sectional genomic and transcriptomic studies failed to identify unifying biological determinants of relapse, we now adopted a longitudinal strategy and performed multi-level omics analyses in 13 matched pairs of initial diagnosis and relapse samples and their corresponding patient-derived xenografts (PDX) models. We extended this sample set by whole-exome sequencing (WES) and methylome analyses in an additional cohort of 25 matched sets of DNA samples obtained from primary patient cells collected at initial diagnosis, remission, and relapse.

## Material and methods

### Patients

The primary cells were obtained from patients recruited in ALL‐BFM 2000, ALL‐BFM‐2009, CoALL97, CoALL03, CoALL09, and ALL‐REZ BFM 2002 trials or from Schneider Children’s Medical Center of Israel from the time points of first diagnosis, remission, and relapse (Supplementary Table 8). Of the 38 patients described here some details of 13 patients were previously reported [3].

Clinical trials from which samples were used in this analysis had previously received approval from the relevant institutional review boards or ethics committees. Written informed consent had been obtained from all the patients or legal guardians, and the experiments conformed to the principles set out in the WMA Declaration of Helsinki and the Department of Health and Human Services Belmont Report.

### Whole-exome and ATAC sequencing

WES and ATAC-Seq were performed as described before [18, 19] - for details see Supplementary Materials.

### Analysis of cancer predisposing genes

We generated a list of 227 potential CPG (Suppl. Tab. 9) by combining previously reported tumor suppressors or genes involved in DNA repair [20–24]. Sixty-two remission samples (day 33) from 38 patients who later developed a relapse and from 24 who did not relapse were subjected to WES. Of the constitutional variants (AF ≥30%) we focused exclusively on: stopgain/loss, frameshift InDels, splicesite donor/acceptor in 227 CPGs.

### DNA methylation analysis using 450k BeadChip Arrays and Infinium^®^ MethylationEPIC BeadChip Arrays

Bisulfite‐conversion, analysis on the Infinium® Methylation assay and EPIC assay (Illumina) and downstream analysis using the RnBeads software package [25] were performed as reported before [19].

### Software and bioinformatical tools

For graphical representation and statistical analyses, GraphPad and R [26] were used. R packages: DESeq2, DNA copy, ggplot2, reshape2, scales.

Graphical abstract was created with BioRender.com.

## Results

### Clonal selection in type-2 T-ALL relapses is coupled with a high degree of heterogeneity and an increase of mutation load at relapse

**Fig. 1:**
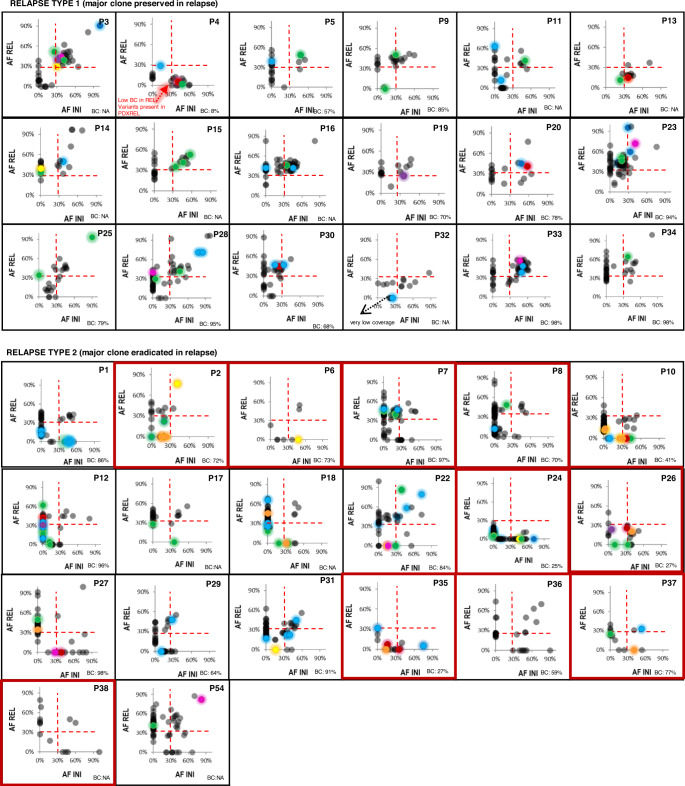
Allele frequency analysis of mutations reveals clonal evolution on the way from initial T-ALL to either type-1 or type-2 relapse. Scatter plots of allele frequency (AF) of mutations detected at initial diagnosis (INI, *x*-axis) and relapse (REL, *y*-axis). Type-1 relapses are defined by all clonal mutations with allele frequencies (AF) > 30% being preserved at relapse; in type-2 relapses, the major clone present at initial diagnosis is eradicated as defined by a subset of clonal mutations with AF > 30% being lost in the relapse. BC = blast content at the time of relapse; dashed red line – 30% allele frequency threshold for clonal (AF ≥ 30%) and subclonal (>30%) mutations; color code: NOTCH1/3 – green, FBXW7 – orange, NRAS/KRAS – red, IL7R/JAK/STAT pathway – yellow, PI3K pathway – violet, ribosomal genes – pink, chromatin modifiers – blue, the remaining mutations are labelled in grey; red frames indicate those patients in whom CNA analysis confirmed type-2 relapse. P4REL – because of the blast content of only 8% the leukemia cells isolated from the relapse, PDX of this patient was used for classification, which demonstrated this relapse to be of type-1; P32 – the mutations lost at relapse had very low coverage; P2 – low blast content at initial diagnosis; the CNA pattern together with the analysis of corresponding PDXs suggests a type-2 relapse.

Based on the profile of single nucleotide variants (SNV) and insertions/deletions (InDels) (Fig. 1) generated by WES of all 38 patients who were analyzed at the time of initial diagnosis, remission, and relapse we distinguished 18 (47%) patients with type-1 relapses as defined by all clonal mutations with allele frequencies (AF) >30% being preserved at relapse. In 20 (53%) patients with type-2 relapses the major clone present at initial diagnosis was eradicated as defined by a subset of clonal mutations with AF >30% being lost in the relapse [3]. CNA/CN-LOH (copy number alterations/copy-neutral loss of heterozygosity) analyses showed a loss of CNA/CN-LOH profiles detected during initial disease in 9/20 type-2 relapses, confirming the loss of the major clone in these patients (Supplementary Fig. 1). By contrast, no losses of CNA/CN-LOH profiles were observed among type-1 relapses.

**Fig. 2:**
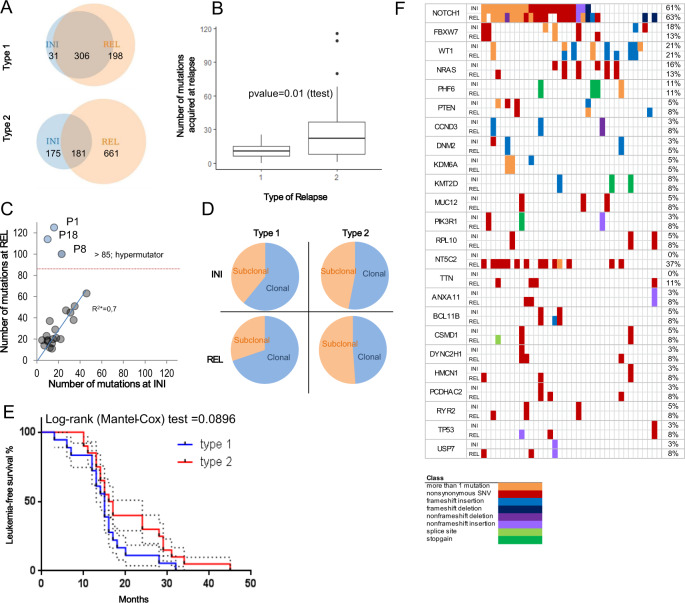
T-ALL type-1 and type-2 relapses are biologically and clinically distinct. **A** Numbers of mutations detected by whole exome sequencing in matched samples collected at initial diagnosis (INI), at relapse (REL) and at both time-points from: 18 T-ALL patients relapsing with type-1 and in 20 T-ALL patients relapsing with type-2. **B** Boxplot presenting numbers of mutations acquired by T-ALLs relapsing as type-1 and type-2. **C** Numbers of mutations detected at initial diagnosis plotted against the number of mutations detected at relapse per patient; 3 hypermutator T-ALLs (P1, P8 and P18) carry >85 mutations at relapse; * - the hypermutators were excluded from the correlation analysis. **D** Proportion of clonal (AF ≥ 30%) and subclonal (AF < 30%) mutations at initial diagnosis and at either type-1 or type-2 relapse. Allele frequencies were not corrected for different blast content in the samples, except for sample collected from P2 at initial diagnosis, where contamination with normal cells was observed (Suppl. Tab. 2). **E** Kaplan–Meier curve showing leukemia-free survival of T-ALL patients following either type-1 (blue line) or type-2 (red line) relapse. **F** Recurrent mutations found by WES in the 38 matched pairs of initial (INI) and relapse (REL) T-ALL patients.

Whereas leukemias relapsing as type-1 preserved most of the coding mutations (306/337; 91%) detected at initial diagnosis, type-2 leukemias preserved only 51% (181/356; Fig. 2A). Moreover, the number of mutations acquired at the time of type-1 relapse was significantly lower when compared to type-2 (type-1: mean ± SEM: 10.94 ± 1.677 *N* = 18; vs. type-2: mean ± SEM: 33.05 ± 7.636 *N* = 20; *p* = 0.01 (ttest, unpaired)) indicating that type-2 T-ALLs underwent stronger genomic remodeling on the way to relapse (Fig. 2B). A substantial increase in the mutational load in type-2 relapses was particularly evident in three patients: P1, P8, P18 (Fig. 2C), who carried more than 85 coding mutations at the time of relapse. This remarkable accumulation of mutations was likely caused by gains of mutations in the Bloom RecQ helicase (*BLM*; P18), simultaneous mutations in the mitotic checkpoint serine/threonine-protein kinase (*BUB1B*) and the mismatch repair endonuclease *PMS2* (P8), and in *TP53* (P1), respectively. P18 (*BLM*) and P8 (*BUB1B/PMS2)* acquired the highest numbers of somatic deletions (Supplementary Fig. 2a), and an analysis of the mutation context suggests a dominant contribution of Cosmic Mutational Signature 6, which is indicative of defective DNA mismatch repair or microsatellite unstable tumors (Supplementary Fig. 2b). Remarkably, the fraction of subclonal mutations of those T-ALLs that later developed into a type-2 relapse was significantly higher already at the time of initial diagnosis than in those T-ALLs that later developed into a type-1 relapse (type-1: 131/337 (39%); type-2: 167/356 (47%), *p* = 0.0387; Fisher’s exact), a difference that became even more pronounced at the time of relapse (type-1: 153/504 (30%); type-2: 431/842 (51%)); *p* < 0.0001; Fisher’s exact; Fig. 2D). These findings suggest fundamental biological differences between these types of T-ALLs already at the time of the initial disease, which become even more apparent at the time of relapse. These divergently developing genomic changes are paralleled by a trend towards a longer time to relapse in type-2 than in type-1 patients (*p* =  0.0896 (Mantel-Cox test); Fig. 2E).

We next analyzed the mutation spectrum (Fig. 2F) in the entire group of 38 patients according to relapse type. The most frequent were *NOTCH1* mutations (*N* = 48) found in 21 patients. Of these only patients relapsing with type-1 preserved all the *NOTCH1* mutations detected with an AF >30% at the time of initial diagnosis (*N* = 9 patients). The majority of the lost (11/12) and gained (12/14) *NOTCH1* mutations were detected in type-2 relapse (Supplementary Table 1). However, this pattern was also observed for variants of other genes thus not implicating *NOTCH1* as a specific driver of either of the types of relapse. Activating mutations of cytosolic 5′-nucleotidase II (*NT5C2*) occurred in 6/18 type-1 and 8/20 type-2 relapses and in none of the initial diagnosis samples, thus confirming the frequent acquisition of *NT5C2* mutations in T-ALL relapses [8–10]. Seven of these mutations were gain-of-function variants at position R367 and two at R238 (for the remaining see Supplementary Tab. 1). Six mutations carried by 5 of these 14 patients (4 type-1 and 1 type-2) were subclonal (AF < 30%). RAS mutations occurred in 4/18 type-1 and in 5/20 type-2 T-ALLs, both at the time of initial and/or relapsed disease. *TP53* mutations occurred at relapse in 1/18 type-1 and in 2/20 type-2 T-ALLs. In one of these patients, the *TP53* mutation was traced back to the initial diagnosis sample where it was detected with a low AF of 5% (3/58 reads). These findings indicate that the leukemogenic mechanisms that are activated by these mutations are shared between both types of relapses.

### Constitutional mutations in cancer predisposition genes are enriched in T-ALL patients with a type-2 relapse

Because of the growing evidence for the role of predisposing constitutional variants that contribute to approximately 10% of all pediatric malignancies [20, 21, 27, 28] we analyzed matched remission samples (collected at day 33 of treatment) for inherited variants in 227 known cancer predisposition genes (CPG) compiled from previously reported studies [20, 22–24, 29] and those analyzed by the PCAWG (pan-cancer analysis of whole genomes) network [30]. In 7/20 type-2 patients we detected nine constitutional heterozygous variants (AF ≥30%) predicted to be deleterious or disruptive for splicing (nonsense- frameshift and splice site mutations) in genes associated with DNA repair (*CHEK2, ERCC2/3, SPRED1, GTF2H3, RFC3, POLR2L*) and in tumor suppressor genes (*VHL*, *FBXW7*). Constitutional mutations in these genes are associated with cancer predisposition syndromes [20–22, 24]. By contrast, such constitutional variants were not identified in any of the type-1 patients (type-1 vs. type-2; 0/18 vs. 7/20; *p* = 0.0087; Fisher's exact test, two-tailed). None of the patients carrying constitutional cancer predisposition mutations exhibited a somatic hypermutator phenotype. In order to assess the role of constitutional CPG mutations (Supplementary Table 8) in conferring a higher risk of relapse we compared the frequency of CPG mutations in remission samples of the relapsing patients with patients who did not develop a relapse. These data did not indicate a significantly difference between these groups. Similarly, a comparison of the frequency of constitutional inactivating mutations in cancer predisposition genes (Supplementary Table 8) between the non-cancer gnomAD cohort (v2.1.1 [31]) and T-ALL patients showed an even distribution (see Supplementary Results for details). These data indicate that CPG mutation shape the evolution of a relapse towards type-2 instead of type-1 but do not increase the overall risk of developing a T-ALL or a T-ALL relapse. We hypothesize that these mutations favor the accumulation of DNA damage under conditions of genotoxic stress such as chemotherapy. Unfortunately, the incompleteness of clinical data and family histories on secondary malignancies precluded a systematic analysis of the association with hereditary cancer predisposition. However, the development of mutational inactivation of a tumor suppressor in a patient with tumor predisposition could be monitored particularly well in P27, who presented with a simultaneous Wilms tumor at the time of initial diagnosis. This patient carried a constitutional previously unknown frameshift mutation of *FBXW7* (Met587ArgfsTer41; chr4:152324250). In T-ALL, inactivation of *FBXW7* is a common cause for activation of *NOTCH1*-pathway and other oncogenic clients such as *MYC* [18]. The sample collected at initial diagnosis of T-ALL exhibited LOH of *FBXW7* in addition to the constitutional frameshift mutation (Supplementary Fig. 3), whereas at the time of relapse that occurred unusually late 913 days after the initial diagnosis this T-ALL had acquired a second, somatic missense mutation (c.1033A > G; p.T345A) in proximity to the known hotspot (R347). Samples of the patient’s parents and siblings were not available, nor was a sample representing the synchronous Wilms tumor. We next asked whether constitutional inactivation of *FBXW7* might be a common predisposing mechanism in T-ALL and screened 51 remission samples of T-ALL patients in whom we had previously found a somatic *FBXW7* mutation [18]. Sanger sequencing of exons 9 and 10 of *FBXW7* did not identify constitutional mutations in any of these patients, indicating that constitutional loss of *FBXW7* function is not common in pediatric T-ALL.

### Transcriptomic and epigenomic profiles of T-ALL are determined by the leukemogenic fusion gene and are preserved between initial diagnosis and relapse

**Fig. 3:**
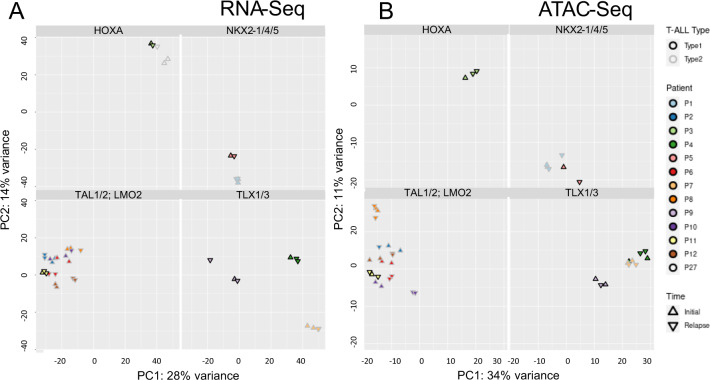
Unsupervised RNA-Seq and ATAC-Seq principal component analyses of initial diagnosis and relapsed T-ALL cluster samples according to the driver fusion. Unsupervised analysis of (**A**) the RNA-Seq (26 samples) based on all genes and (**B**) ATAC-Seq (24 samples) based on all peaks in matched samples of initial diagnosis (circle) and relapse (triangle) collected from 13/12 T-ALL patients (color code). Samples collected from the same patient at different time-points cluster in close vicinity. Most of the variance (RNA-Seq: PC1: 28%, PC2: 14%; ATAC-Seq: PC1: 34%, PC2: 11%) can be explained by aberrant expression of driving fusion genes such as basic helix-loop-helix (bHLH) family members *TAL1*, *TAL2*, LIM-only domain (LMO) gene family or the homeobox genes *TLX1*, *TLX*3, *NKX2-4*, *NKX2-5; HOXA*.

Of the total set of 38 matched pairs of initial diagnosis and relapse, we expanded matched samples of 13 patients in PDX thus enabling us to obtain suitable material (RNA and leukemic cells) for multi-omic analyses (Supplementary Table 2). Of the 12 patients with available mRNA data, 5 were classified as cortical T-ALL, two as mature, two as pre-T, one as pro-T and two as a mixed cortical-T/pre-T (detailed FACS and immunophenotype profiles in Supplementary Table 3). None of the patients was classified as early thymic progenitor (ETP)-ALL, however at the time of diagnosis of some of the patients the high-risk ETP-ALL subtype had not yet been described. Moreover, some expression features resembled those of early thymocytes by high expression of *CD34*, *SPI1* or *KIT* (Supplementary Fig. 4, Supplementary Table 3). Five of 13 patients developed a type-1 and 8 a type-2 relapse. As previously shown directly, early passages of T-ALL PDXs that were used here preserve the genomic and epigenomic profiles and the subclonal architecture of the primary patients’ leukemias with high fidelity [19, 32]. Based on ectopic expression of known T-ALL drivers, we classified this subset of 13 patients into the following subgroups: *TAL1/2* (*n* = 5), *TLX1/3* (*n* = 3), HOXA (*n* = 2), *NKX2‐4/5* (*n* = 2), and *LMO2* (*n* = 1; Fig. 3A). Unsupervised learning approaches clustered all 26 samples (obtained at diagnosis and relapse) according to the driving fusion indicating that these are the strongest factors determining the transcriptomic (Fig. 3A), methylation (Supplementary Fig. 5) and chromatin accessibility profiles (Fig. 3B). At the same time, the overall gene expression, methylation, and chromatin accessibility profiles of the initial and relapsed T-ALLs of individual patients were largely retained following the evolution to relapse (Fig. 3; Supplementary Fig. 5). This similarity is also reflected by differential transcriptomic analyses between initial diagnosis and relapse identifying only 0.13% (43/32 529) genes to be significantly up- or downregulated at relapse (DESeq2; padj <0.05; Supplementary Tab. 4a/b). Similarly, analyses by ATAC-Seq revealed significant changes of the chromatin accessibility profile in only 0.53% (557/105 136) of the analyzed ATAC-peaks (DESeq2, padj < 0.05; Supplementary Table 4c/d). Likewise, significant changes on the way to relapse were neither identified in promoter methylation profiles (RnBeads [25]; Supplementary Table 5). The methylation data were further used for classification into either CIMP+ or CIMP– as described previously [33] (Supplementary Fig. 6). These analyses demonstrate that (1) the CIMP status does not change during the transition from initial diagnosis to relapse in majority of the patients (19/23) and (2) does not differ in its distribution between type-1 (5 CIMP–; 6 CIMP+) and type-2 relapses (8 CIMP–; 5 CIMP+). These data indicate that in a cross-sectional or longitudinal approach not considering the type of relapse any relapse-specific alterations in the transcriptomic and epigenomic profiles are masked by the interpatient heterogeneity and by the dominant fusion genes driving the leukemogenesis.

### Relapse specific differences at the epigenomic and transcriptomic level develop in type-2 relapses while type-1 relapses resemble the profiles of the initial disease

**Fig. 4:**
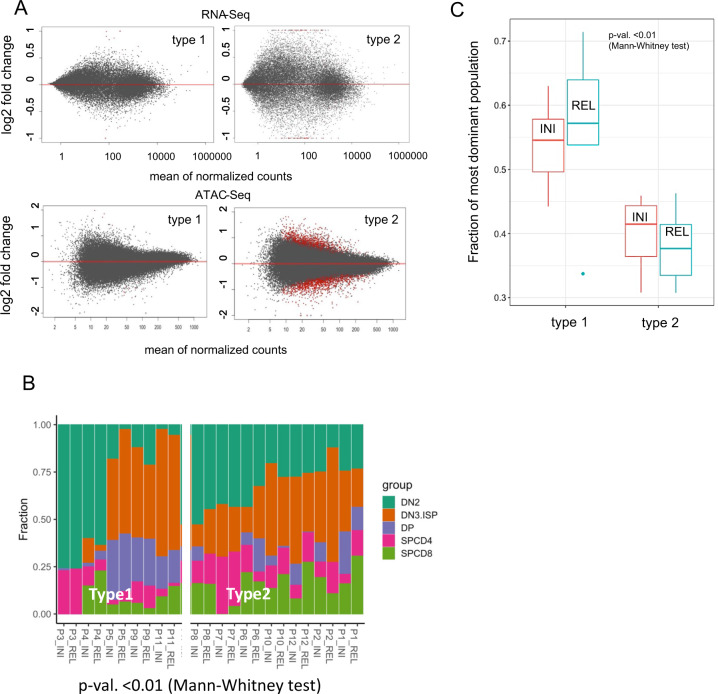
T-ALL type-2 relapses have undergone stronger chromatin remodeling and transcriptional changes than type-1 relapses. **A** Differential analyses (DESeq2, adj *p* < 0.05) of RNA-Seq and ATAC-Seq datasets comparing initial diagnosis and relapse within type-1 and type-2 leukemias. MA plots show log2 fold changes in expression/accessibility in relation to the mean of normalized counts per gene/peak. Significantly differential expressed genes/accessible ATAC-peaks are labeled in red. **B** Deconvolution analysis of T-ALL samples performed with CIBERSORT trained on the signature of 2823 open chromatin regions selected to distinguish five differentiation stages (DN2, DN3/ISP, DPCD3–/ DPCD3+, CD4+, CD8+) of healthy T-cell precursors as previously reported [35] and applied to decompose T-ALL ATAC-profiles. **C** Fraction of the dominant population as predicted by deconvolution with CIBERSORT in T-ALLs relapsing either as type-1 or type-2.

We next tested whether relapse-specific changes may be unmasked when separately analyzing leukemias that developed into either a type-1 or a type-2 relapse. As detailed above for the entire set of 38 patients, we confirmed in 13 matched pairs of initial diagnosis and relapse expanded in PDX-mice that type-2 relapses acquired significantly more mutations (*p* = 0.01 (*t* test, unpaired), Fig. 2A) and are characterized by a higher degree of subclonal complexity than type-1 relapses (Fig. 2D). The analysis of the multi-omics set of 13 patients revealed that the stronger remodeling on the way to type-2 than to type-1 relapses was also reflected by a more complex evolution of epigenetic changes in type-2 relapses. Specifically, when analyzing the methylation profile of the promoters, we observed an almost 10-fold higher mean difference in β value when comparing the initial disease and relapse in type-2 (0.002) with type-1 (0.00034) (*p* < 0.0001, Chi-square test). When analyzing chromatin accessibility, the number of differentially accessible ATAC-peaks was only 14 (0.013%) in type-1 but 852 (0.81%) in type-2 (*p* < 0.0001, Chi-square test; Fig. 4A). A similar difference was observed at the transcriptomic level with only 1 gene being differentially expressed in type-1 and 152 genes in type-2 (*p* < 0.0001, Chi-square test; Fig. 4A, Supplementary Table 6).

We next mapped the T-ALLs to the most closely related maturation stages of thymic T-cell precursors by employing recently reported profiles of chromatin accessibility obtained by ATAC-Seq [19, 34]. Thus, we trained the CIBERSORT algorithm [35] on the signature of 2,823 chromatin regions selected to distinguish five differentiation stages (DN2, DN3/ISP, DPCD3–/ DPCD3+, CD4+, CD8+) of healthy T-cell precursors [34]. The signature regions differentially accessible in maturation stages were enriched for binding motifs of transcription factors that are highly expressed in T-cell precursors, which indicated a match between chromatin accessibility (ATAC-Seq) and gene expression (RNA-Seq). Deconvolution analyses of the T-ALLs obtained at the time of first diagnosis and at relapse revealed that at relapse and even at the time of first diagnosis type-1 leukemias were more homogeneous predominantly resembling a single immature normal T-cell population, whereas the pattern of the type-2 leukemias was more complex resembling a mixture of healthy T-cell precursor populations already at the time of the initial disease (Fig. 4B, *p* < 0.01, Mann-Whitney test; Fig. 4C). These data show that relapse type-specific differences at the epigenomic and transcriptomic level are already apparent at the time of first diagnosis and that relapse-specific changes largely develop in type-2 relapses, whereas type-1 relapses are essentially identical to the profiles of the initial disease.

### Profiles of disease progression and drug resistance in type-1 and type-2 T-ALLs

**Fig. 5:**
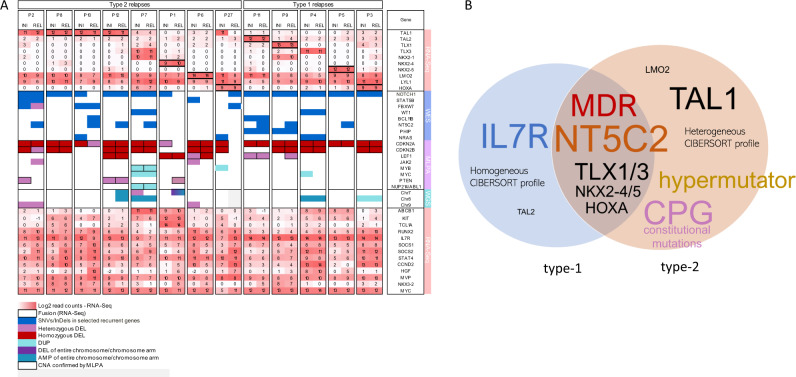
Models of disease progression and drug resistance in type-1 and type-2 T-ALLs are revealed by multi-omics analyses. **A** Molecular characteristics of initial diagnosis (INI) and relapse (REL) of matched samples from 13 pediatric T-ALL patients characterized by RNA-Seq (log 2 transformed read counts; read count values of 0 were not transformed; black frame indicates that fused-transcripts were detected), WES (SNVs/InDels), CNAs in targeted T-ALL specific genes and low coverage whole genome sequencing (large CNAs). Displayed are the most recurrent changes and specific hallmarks of each leukemia. **B** Biological features and potential mechanisms of relapse shared by T-ALLs relapsing either as type-1 or as type-2, and those specific to each relapse type. The font is scaled according to the frequency of the feature.

We next interrogated the epigenomic and transcriptomic data sets of the 13 T-ALLs that could be propagated in xenografted mice for shared and differential profiles in the 5 type-1 and the 8 type-2 T-ALLs (the molecular profiles are summarized in Fig. 5A). We performed differential analyses of the mutation spectrum, RNA-Seq and ATAC-Seq data (DESeq2, *p* < 0.05) of the initial disease and relapse samples both globally and separately for each of the 13 patients (Suppl. Tab. 7). In 4/13 patients (2 type-1 and 2 type-2) we detected increased mRNA expression (Supplementary Table 4a) of multidrug resistance gene 1 *MDR1 (ABCB1)* both at the time of initial disease and relapse. In another two patients we observed an at least two-fold upregulation of the *MDR1* gene expression at relapse (Fig. 5A; Supplementary Table 4a). *MDR1* is known to confer in vitro resistance to many drugs used in the treatment of acute leukemia such as the anthracyclines, vincristine, vinblastine, and methotrexate [36]. Additionally, we identified a relapse-specific 6-fold upregulation of the major vault protein (*MVP*) gene in two patients whose relapse was classified as type-2. In patient P2 the *MVP* normalized read count increased from initial disease to relapse from 138 to 800 and in P10 from 409 to 2464 (mean of all patients: 594; median: 416; SD: 493). *MVP* is involved in nucleocytoplasmic transport and has been reported to be overexpressed in multi-drug resistant solid tumors [37]. These findings, together with the recurrent gain of function mutations of *NT5C2* reported previously and shown above, indicate that both, in type-1 and type-2 T-ALL relapses, expression of drug resistance genes contribute to the frequent treatment resistance and poor prognosis. In the set of 13 initial disease/relapse pairs *TAL1*-driven T-ALLs were exclusively identified in those patients developing type-2 relapses (4/8 type-2 vs. 0/5 type-1; *p* = 0.1; Fisher's exact test, two-tailed) suggesting that TAL1 may play a specific role in type-2 T-ALL relapses (Fig. 3, Fig. 5), although clearly this trend will have to be validated in a larger number of samples. Functional enrichment analysis of the most recurrently upregulated genes (*N* > 5) showed enrichment in the regulation of the JAK-STAT cascade. At relapse, 11 of the 13 patients show upregulation of at least one of the *SOCS1/2/3*, which regulate the JAK-STAT pathway via negative-feedback loop confirming previous reports [38–40] that the activation of this protooncogenic pathway is common in T-ALL. Notably, however, overexpression of the *IL7R* gene was limited to the 5 patients with type-1 relapses, only in one it was correlated with the presence of an activating *IL7R* gene mutation. In addition to the overexpression of the *IL7R* itself, the functional role of this pathway was indicated by the overexpression of other genes involved in its regulation and by the overexpression of *MYC*, the cell cycle regulator cyclin D2 and the anti-apoptotic member of the Bcl-2 family *MCL1* (Supplementary Fig. 7).

## Discussion

Because most intensive chemotherapy regimens in pediatric T-cell leukemia have reached the limit of tolerability, current research efforts are focusing on stratifying treatment intensity according to risk. While several genetic parameters determining risk are known for precursor B-ALLs [41], risk stratification of T-ALL is still largely based on the kinetics of treatment response [42]. Furthermore, there are currently no established specific molecular targets in T-ALL which can be exploited for therapy. Patients with T-ALL are therefore likely to particularly benefit from a detailed understanding of the biology of the disease and the mechanisms that govern relapse.

We have previously reported that T-ALL relapses can be classified as either type-1, characterized by the major clone present at the time of initial disease evolving by acquiring additional mutations, or type-2, which is characterized by the major clone of the initial disease having been eradicated and the relapse having evolved from a minor ancestral clone [3]. Approximately half of the T-ALL patients who suffer from a relapse can be classified as either type-1 or type-2, respectively. We show here that on the path from initial disease to type-1 relapse, T-ALLs tend to remain stable in their mutational spectrum, promoter methylation, chromatin accessibility and gene expression profile. In contrast, type-2 T-ALL relapses are characterized by intensive remodeling as indicated by an increase in mutational load, changes in DNA methylation, chromatin accessibility and in gene expression. It is important to note that these differences only became apparent when patients were classified according to their disease evolution during progression, whereas they remained masked when analyzing type-1 and type-2 T-ALL relapses together. It has been an unexpected finding, that fundamental differences in genomic, epigenomic and transcriptomic profiles between T-ALLs that will relapse as either type-1 or type-2 are already apparent at the time of first diagnosis. Specifically, T-ALLs later evolving into type-1 relapses show a more homogeneous subclonal architecture and chromatin maturation profiles. An important finding of this study is that constitutional mutations in tumor suppressor genes and in genes involved in DNA repair likely contribute to the genetic instability and accumulation of mutations of leukemias that develop into type-2, but not type-1 relapses. While the analysis of probands reported not to develop malignancies or of T-ALL patients who did not develop a relapse showed that the carrier status for these mutations is neither associated with a significantly increased risk of developing a T-ALL nor of a T-ALL relapse, these mutations implicated in cancer predisposition, likely shape the type of relapse following exposition to leukemogenic stimuli and to genotoxic treatment. Type-1 relapses are characterized by early progression and occur in leukemias that already at the time of initial diagnosis are equipped with mechanisms driving treatment resistance. This is particularly apparent in three patients (P13, P25, and P32), who not only preserved almost all the mutations detected at initial diagnosis but did not acquire additional mutations at the time of relapse. Type-2 relapses, by contrast tend to develop later, the leukemia requiring more extensive genetic and epigenetic remodeling of a subclone of the initial disease, a process that appears to be facilitated by constitutional mutations in cancer predisposition genes. It must be noted that the genomic analyses performed here do not fully explain all differences between T-ALL at diagnosis and at relapse. Specifically, some of the type-2 relapses display a substantially different spectrum of variants when compared to the samples obtained at the time of initial diagnosis. By contrast, these samples do not show equally substantial differences at the epigenomic and transcriptomic level. This observation suggests that many variants may not or only marginally contribute to the driving of the leukemia. Future single cell analyses may provide insight into the specific role of some variants to the biology of the leukemia. With the analysis of a larger number of matched leukemia pairs, we expect that our classification may be updated to a version with more subtypes, for example possibly specifying those that have neither gained or lost mutations during the transition from diagnosis to relapse.

The type-specific and longitudinal analysis of individual patients we performed here revealed shared and type-specific profiles that develop during the transition from initial disease to relapse (Fig. 5B). Both types of relapse share the recurrent acquisition of *NT5C2* mutations at relapse, therefore confirming previous reports implicating this mutation as a common relapse-specific mechanism of drug resistance [8–10]. In addition to *NT5C2*, drug resistance has likely been induced in two type-1 and in two type-2 patients with high expression of *MDR1*. Overexpression of this gene has previously been reported to cause resistance to drugs used in the treatment of acute leukemia via ATP-dependent drug efflux [36]. At the time of relapse, two further patients exhibited an increased expression of the *MVP* mRNA, a gene that is implicated in drug resistance in solid tumors [43, 44] and which may thus play a previously unrecognized role in T-ALL. Taken together, these data indicate that the activity of multidrug resistance genes likely contributes to the poor prognosis of both types of T-ALL relapses.

When considering type-specific differences, overexpression of *IL7R* emerged as a characteristic feature of type-1 leukemias, remarkably both at the time of relapse and already at the time of first diagnosis. While only one of the patients analyzed here carries an activating *IL7R* mutation, we observed a possibly compensatory upregulation of components of the IL7R-JAK-STAT pathway: its ligand *HGF*, *IL7R* itself and of suppressors of cytokine signaling (*SOCS1*, *SOCS2*, *SOCS3*), which regulate the pathway via a negative-feedback loop. Suppressors of cytokine signaling were previously identified as part of a stemness-related molecular signature signifying unfavorable outcome in acute myeloid and lymphoblastic leukemia [45]. Gain-of-function mutations in interleukin-7 receptor-α conferring cytokine-independent growth of progenitor lymphoid cells were first described by Shochat and colleagues [46, 47] and shown to be involved in human T-cell leukemogenesis [48] and drug resistance in T-ALL [49]. Although analyses in the UK ALL2003 trial showed that an activation of IL7R-JAK did not confer an adverse prognosis in T-ALL [50], in vitro analysis of steroid resistance showed an association of IL7R-JAK-STAT mutations with a strong activation of the downstream oncogenic MEK-ERK and AKT pathways; thereby inducing a robust antiapoptotic response by upregulating *MCL1* and *BCLXL* expression [49]. Moreover, recent studies in pediatric B-cell precursor (BCP) acute lymphoblastic leukemia linked high expression of *IL7R* with CNS infiltration and relapse [51]. Previous reports suggest that overexpression of *IL7R* and the activation of downstream targets may represent therapeutic targets of either specific antibodies or small molecule inhibitors such as ruxolitinib [51, 52]. However, overexpression alone of molecular targets has recently emerged not to reliably predict treatment response by targeted therapy in pediatric malignancies [53]. Irrespective of its potential role as a molecular target, the results reported here implicate activation of the IL7R pathway as a leukemogenic event that preferentially occurs in type-1 T-ALL.

In conclusion, the data presented here show that the analysis of relapse-specific mechanisms in T-ALL is substantially facilitated by analyzing type-1 and type-2 relapses separately. Specifically, this work shows shared and type-specific profiles that are already apparent at the time of diagnosis. These profiles include the frequent upregulation of the IL7R pathway in type-1 and the enrichment of constitutional cancer predisposition genes and hypermutator phenotypes in type-2 relapses.

## Supplementary information

Suppl. Tab. 1

Suppl. Tab. 2

Suppl. Fig. 3

Suppl. Fig. 4

Suppl. Tab. 5

Suppl. Tab. 6

Suppl. Tab. 7

Suppl. Tab. 8

Suppl. Tab. 9

Suppl. Fig. Legends

Supplemental Material

Suppl. Fig. 1

Suppl. Fig. 2

Suppl. Fig. 3

Suppl. Fig. 4

Suppl. Fig. 5

Suppl. Fig. 6

Suppl. Fig. 7

## Data Availability

Sequence data are deposited at the European Genome‐phenome Archive (http://www.ebi.ac.uk/ega/) under accession number EGAS00001003248. Scripts for the plots and tables are available in a repository (https://github.com/tobiasrausch/t-all).

## Code availability

The code for RNA & ATAC alignments, peak calling, read counting etc. is available on GitHub (https://github.com/tobiasrausch/ATACseq, https://github.com/tobiasrausch/RNAseq). For further information see Suppl. Materials.
