# Supplementary material for: Pediatric T-ALL type-1 and type-2 relapses develop along distinct pathways of clonal evolution
Source: Leukemia. 2022 May 18;36(7):1759–68. doi: 10.1038/s41375-022-01587-0 (PMC9252914; doi:10.1038/s41375-022-01587-0)
Supplement: Supplementary file 10 — Suppl. Fig. Legends [file 41375_2022_1587_MOESM10_ESM.docx]

**Suppl. Fig. 1**

Copy number alterations detected in WES of 38 matched pairs of initial diagnosis and relapse samples; shades of violet - large deletion (entire chromosome/chromosome arm)/middle-size deletion/small deletion; shades of red - large amplification (entire chromosome/chromosome arm)/middle-size amplification/small amplification, shades of green - large CN-LOH (entire chromosome/chromosome arm)/middle-size CN-LOH/small CN-LOH.

**Suppl. Fig. 2**

Size of deletions and insertions per patient as analyzed by WES (A); mutational patterns of different substitutions: C>A; C>G; C>T; T>A, T>G; T>C; (B) P8 and P18 show a dominant contribution of Cosmic Signature 6, which is indicative of defective DNA mismatch repair or microsatellite unstable tumors.

**Suppl. Fig. 3**

LOH of the FBXW7 gene in patient P27. The coverage of the FBWX7 gene on the long arm of chromosome 4 (4q31.3) is shown for initial diagnosis, relapse and remission by IGV.

**Suppl. Fig. 4**

Heatmap showing the expression profile (z-scores) of the initial diagnosis and relapse from the 13 T-ALL patients expanded as PDX of the genes differentially expressed in ETP-ALL (Coustan-Smith E et al; Lancet Oncol. 2009).

**Suppl. Fig. 5**

Unsupervised hierarchical clustering (A) based on the average degree of methylation of the 500 most variable promoters (red—low/violet—high methylation levels); * relapse (REL) sample of patient P4 with blast content of 8%.

**Suppl. Fig. 6**

Unsupervised Euclidean hierarchical clustering was performed using beta values for the 1205 of the 1347 most variable CpG sites described before^32^. High methylation levels are shown in red and low levels in green, according to the Beta value scale bar in the figure where 1.0 is fully methylated and 0 is unmethylated.

**Suppl. Fig. 7**

Scatter plot showing correlation between the normalized read counts for *IL7R* (y-axis) and the normalized read counts of *MYC* (orange) and cell cycle regulators *MCL1* (blue) and cyclin D2 (grey).
