# Supplementary material for: Pediatric T-ALL type-1 and type-2 relapses develop along distinct pathways of clonal evolution
Source: Leukemia. 2022 May 18;36(7):1759–68. doi: 10.1038/s41375-022-01587-0 (PMC9252914; doi:10.1038/s41375-022-01587-0)
Supplement: Supplementary file 11 — Supplemental Material [file 41375_2022_1587_MOESM11_ESM.docx]

# SUPPLEMENTARY RESULTS

## Constitutional mutations in cancer predisposition genes are enriched in leukemias relapsing with type-2

In order to assess if constitutional mutations in the genes listed in Suppl. Tab. 9 generally confer a higher risk of relapse in T-ALL, we analyzed remission samples of pediatric T-cell leukemia patients who did not develop a relapse. We identified such constitutional heterozygous mutations in four of 24 patients (in *PARP4, POLQ, OGG1, XPA*), indicating that such mutations do not significantly increase the overall risk of relapse (p=0.75 in comparison to patients with a relapse, Fisher's exact test).

Next, we asked if constitutional inactivating mutations in cancer predisposition genes (Suppl. Tab. 9) are associated with a higher risk of pediatric T-ALL in general. Among the non-cancer gnomAD cohort (v2.1.1^1^) 15% carried such mutations (p=0.273 in comparison to patients with T-ALL relapse; Fisher's exact test; for details, see Suppl. Methods). The even distribution of such mutations among non-cancer patients and patients with T-ALL indicates that they do not generally increase the risk of T-ALL, but rather shape the evolution of a relapse towards type-2 instead of type-1. We hypothesize that these mutations favor the accumulation of DNA damage under conditions of genotoxic stress such as chemotherapy.

# SUPPLEMENTARY METHODS

## Patients

Of the 38 patients described here some details of 13 patients were previously reported (P2=A61, P6=F110, P9=T92, P13=S00169, P14=S00438, P15=S00456, P16=S00207, P17=S00472, P18=S00285, P27=E114, P32=KI17, P36=MD40, P38=T128)^2^.

## Establishment of the patient‐derived xenografts

Patient‐derived xenografts were generated as described^3^ by intrafemoral injection of 1 × 10^5^ to 5 × 10^6^ viable primary ALL cells in NSG (NOD.Cg‐PrkdscidIl2rgtm1Wjl/SzJ) mice. Transplanted mice were both male and female, aged 5–8 weeks. Animals were housed in individually ventilated cages with access to food and water ad libitum. Leukemia progression was monitored in the peripheral blood by flow cytometry using anti‐mCD45, anti‐hCD45, anti‐hCD19, or anti‐hCD7 antibodies. Cells were harvested after engraftment had reached 75% in the peripheral blood or the mice health score had reached either three at single item or the total score had reached five. T‐ALL cells were collected from spleen and cryopreserved as described^3^. Leukemia progression was monitored in the peripheral blood by flow cytometry using anti-mouse-CD45 (anti-mCD45), anti-human-CD45 (anti-hCD45), anti-hCD19, or anti-hCD7 antibodies. Xenograft identity was verified by DNA fingerprinting using the commercial AmpFlSTR® NGM Select kit. In vivo experiments were approved by the veterinary office of the Canton of Zurich, in compliance with ethical regulations for animal research.

## Whole-exome sequencing

Libraries for whole‐exome sequencing were prepared with SureSelectXT Target Enrichment System for Illumina Paired‐End Multiplexed Sequencing Library v4/v6 (Agilent, Santa Clara, CA, USA) according to the manufacturer's protocols and sequenced in paired‐end 100‐bp mode using an Illumina HiSeq2000 deep sequencing instrument (Illumina, San Diego, CA, USA). For variant detection, the union of SNVs/InDels identified by mutation callers Mutect^4^, Strelka^5^ and FreeBayes^6^ was generated. All detected variants were manually curated using the Integrated Genome Viewer. The presence of leukemia-specific variants in the germline was excluded by analysis of a matched remission sample. We have filtered all variants detected with an allele frequency < 5% unless they were present at both initial diagnosis and relapse of the same patient or showed more than 5 supporting reads.

The estimated blast content failed to serve as a valid parameter to estimate allele frequencies in leukemia cells, because the allele frequencies calculated on that basis exceeded 100% in some samples. Therefore, the allele frequency was adjusted, by employing FACETS^7^ to estimate sample purity by allele-specific copy number analysis (Suppl. Tab. 1). Correlation between the blast content and the calculated purity was high (R^2^=0.819). Nevertheless, for app. 30% of the samples for which both estimates were available we observed that FACETS estimates were 1.5-4.8 times lower than the estimated blast content. These underestimates were not in agreement with the observed AF of the mutations in these samples and were probably caused by the absence of clonal CNAs. Thus, to make sure that the impurity of the samples did not affect the classification into type 1 and type 2 relapses we have carefully inspected depth of the matched initial diagnosis and relapse samples at the mutated position (Suppl. Tab. 1) instead of correcting for the blast content/sample impurity. In P4 the blast count at the time of relapse was too low (8%) to reliably detect variants, and therefore the PDX sample originating from the relapsed leukemia was used for comparison with the sample from the time of initial diagnosis. This analysis resulted in P4 being classified as a type-1 relapse.

## ATAC-Seq

Briefly, we used 50,000 cells and permeabilized the cell membranes with digitonin to reduce the contamination by mitochondrial reads (for details see^8^). DNA libraries were sequenced on Illumina platforms (NextSeq500) and data analysis was carried out using an in‐house developed ATAC‐Seq pipeline (<https://github.com/tobiasrausch/ATACseq>) as described before^8^.

## Analysis of cancer predisposing genes

To analyze the populational frequencies in the gnomAD database (v2.1.1^1^) we selected a cohort of 134,187 non-cancer patients and calculated the likelihood of having a protein truncating variant with a population allele frequency <0.01% in one of the CPGs (Suppl. Tab. 9) by dividing the number of variants by the total number of allele counts.

## RNA-Seq

# Total RNA was extracted using TRIzol (Invitrogen Life Technologies). RNA was then treated with TURBO DNase (Thermo Fisher Scientific, Darmstadt, Germany) and purified using RNA Clean&Concentrator-5 (Zymo Research, Freiburg, Germany). A minimal RIN (RNA Integrity Number) of 7 as measured using Bioanalyzer (Agilent, Santa Clara, CA) with Agilent RNA 6000 Nano Kit was required for the sample to be sequenced. Cytoplasmic ribosomal RNA was depleted by Ribo-Zero rRNA Removal Kit (Illumina, San Diego, CA) and the libraries were prepared from 1 µg of RNA using TruSeq RNA Library Prep (Illumina, San Diego, CA) at Genomics Core Facility of the EMBL, Heidelberg. Six RNA samples were pooled and sequenced on one Illumina HiSeq 2000 lane in 75 bp single-end modus. Adapters were removed using cutadapt^9^. Only sequencing reads preferentially aligning to hg19 were further aligned using STAR aligner^10^. Gene fusion discovery in RNA-Seq data was performed using Arriba^11^ and deFuse^12^ algorithms. Mean of read counts per gene was calculated for the samples for which biological replicates (different mice engrafted with the same T-ALL material) were available (14/26). Differential expression analysis between initial diagnosis and relapse samples was performed using DESeq2^13^ combined with fold-change analyses (due to insufficient number of biological replicates required for DESeq2 in 2 of the cases).

1 Karczewski KJ, Francioli LC, Tiao G, Cummings BB, Alföldi J, Wang Q *et al.* The mutational constraint spectrum quantified from variation in 141,456 humans. *Nature 2020 581:7809* 2020; **581**: 434–443.

2 Kunz JB, Rausch T, Bandapalli OR, Eilers J, Pechanska P, Schuessele S *et al.* Pediatric T-cell lymphoblastic leukemia evolves into relapse by clonal selection, acquisition of mutations and promoter hypomethylation. *Haematologica* 2015; **100**: 1442–1450.

3 Schmitz M, Breithaupt P, Scheidegger N, Cario G, Bonapace L, Meissner B *et al.* Xenografts of highly resistant leukemia recapitulate the clonal composition of the leukemogenic compartment. *Blood* 2011; **118**: 1854–1864.

4 do Valle ÍF, Giampieri E, Simonetti G, Padella A, Manfrini M, Ferrari A *et al.* Optimized pipeline of MuTect and GATK tools to improve the detection of somatic single nucleotide polymorphisms in whole-exome sequencing data. *BMC Bioinformatics 2016 17:12* 2016; **17**: 27–35.

5 Saunders CT, Wong WSW, Swamy S, Becq J, Murray LJ, Cheetham RK. Strelka: Accurate somatic small-variant calling from sequenced tumor-normal sample pairs. *Bioinformatics* 2012; **28**: 1811–1817.

6 Garrison E, Marth G. Haplotype-based variant detection from short-read sequencing. 2012.

7 Shen R, Seshan VE. FACETS: allele-specific copy number and clonal heterogeneity analysis tool for high-throughput DNA sequencing. *Nucleic Acids Research* 2016; **44**: 131.

8 Erarslan‐Uysal B, Kunz JB, Rausch T, Richter‐Pechańska P, Belzen IA, Frismantas V *et al.* Chromatin accessibility landscape of pediatric T‐lymphoblastic leukemia and human T‐cell precursors. *EMBO Molecular Medicine* 2020; **12**. doi:10.15252/emmm.202012104.

9 Martin M. Cutadapt removes adapter sequences from high-throughput sequencing reads. *EMBnet.journal* 2011; **17**: 10–12.

10 Dobin A, Davis CA, Schlesinger F, Drenkow J, Zaleski C, Jha S *et al.* STAR: Ultrafast universal RNA-seq aligner. *Bioinformatics* 2013; **29**: 15–21.

11 Uhrig S, Ellermann J, Walther T, Burkhardt P, Fröhlich M, Hutter B *et al.* Accurate and efficient detection of gene fusions from RNA sequencing data. *Genome Research* 2021; **31**: gr.257246.119.

12 McPherson A, Hormozdiari F, Zayed A, Giuliany R, Ha G, Sun MGF *et al.* deFuse: An Algorithm for Gene Fusion Discovery in Tumor RNA-Seq Data. *PLOS Computational Biology* 2011; **7**: e1001138.

13 Love MI, Huber W, Anders S. Moderated estimation of fold change and dispersion for RNA-seq data with DESeq2. *Genome Biology 2014 15:12* 2014; **15**: 1–21.
