# Supplementary material for: Pediatric T-ALL type-1 and type-2 relapses develop along distinct pathways of clonal evolution
Source: Leukemia. 2022 May 18;36(7):1759–68. doi: 10.1038/s41375-022-01587-0 (PMC9252914; doi:10.1038/s41375-022-01587-0)
Supplement: Supplementary file 12 — Suppl. Fig. 1 [file 41375_2022_1587_MOESM12_ESM.pdf]

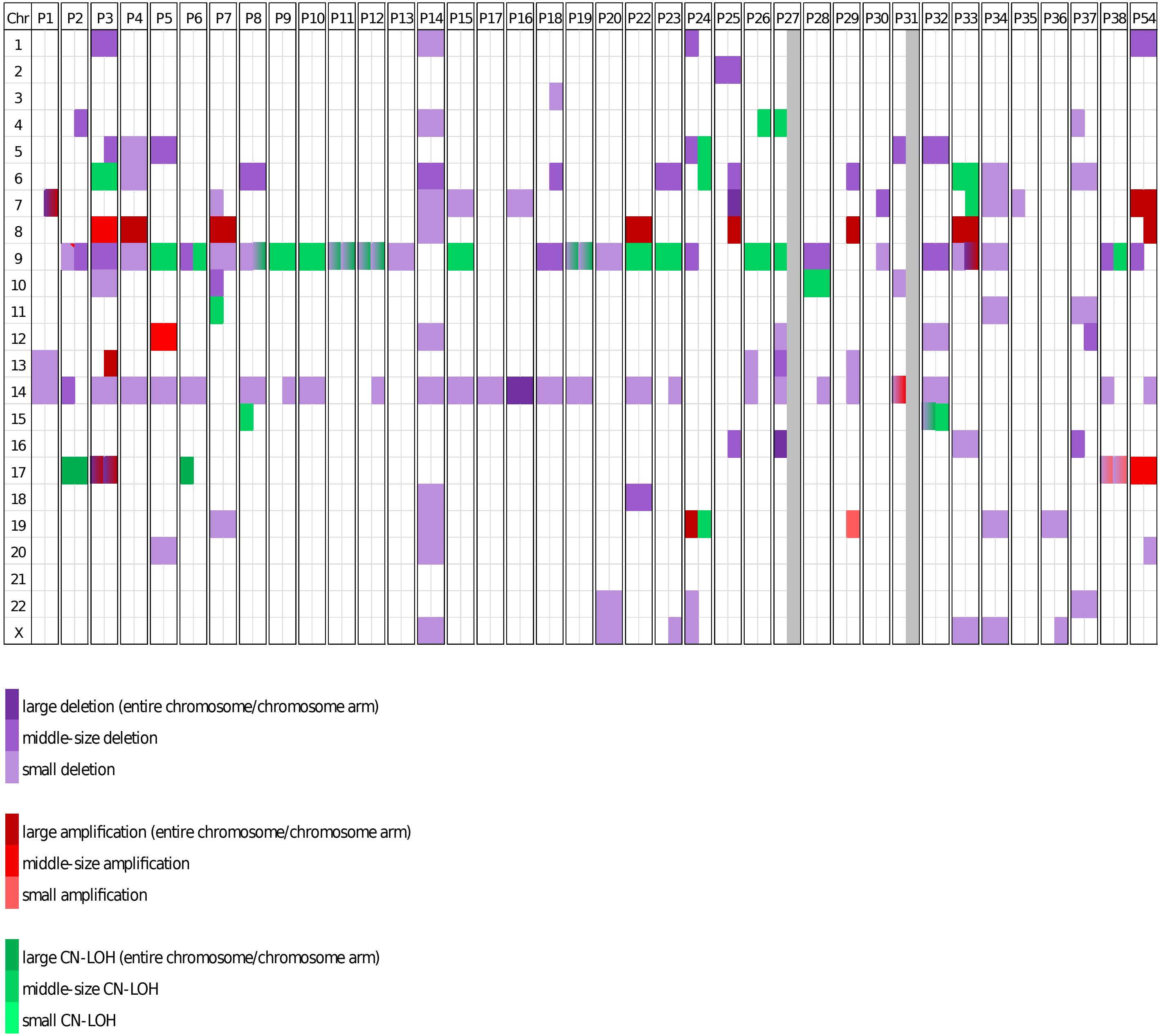
Chr
P1
P2
P3
P4
P5
P6
P7
P8
P9
P10
P11
P12
P13
P14
P15
P17
P16
P18
P19
P20
P22
P23
P24
P25
P26
P27
P28
P29
P30
P31
P32
P33
P34
P35
P36
P37
P38
P54
1
2
3
4
5
6
7
8
9
10
11
12
13
14
15
16
17
18
19
20
21
22
X
large deletion (entire chromosome/chromosome arm)
middle-size deletion
small deletion
large amplification (entire chromosome/chromosome arm)
middle-size amplification
small amplification
large CN-LOH (entire chromosome/chromosome arm)
middle-size CN-LOH
small CN-LOH
