# Supplementary figures and images for: Pediatric T-ALL type-1 and type-2 relapses develop along distinct pathways of clonal evolution
Source: Leukemia. 2022 May 18;36(7):1759–68. doi: 10.1038/s41375-022-01587-0 (PMC9252914; doi:10.1038/s41375-022-01587-0)

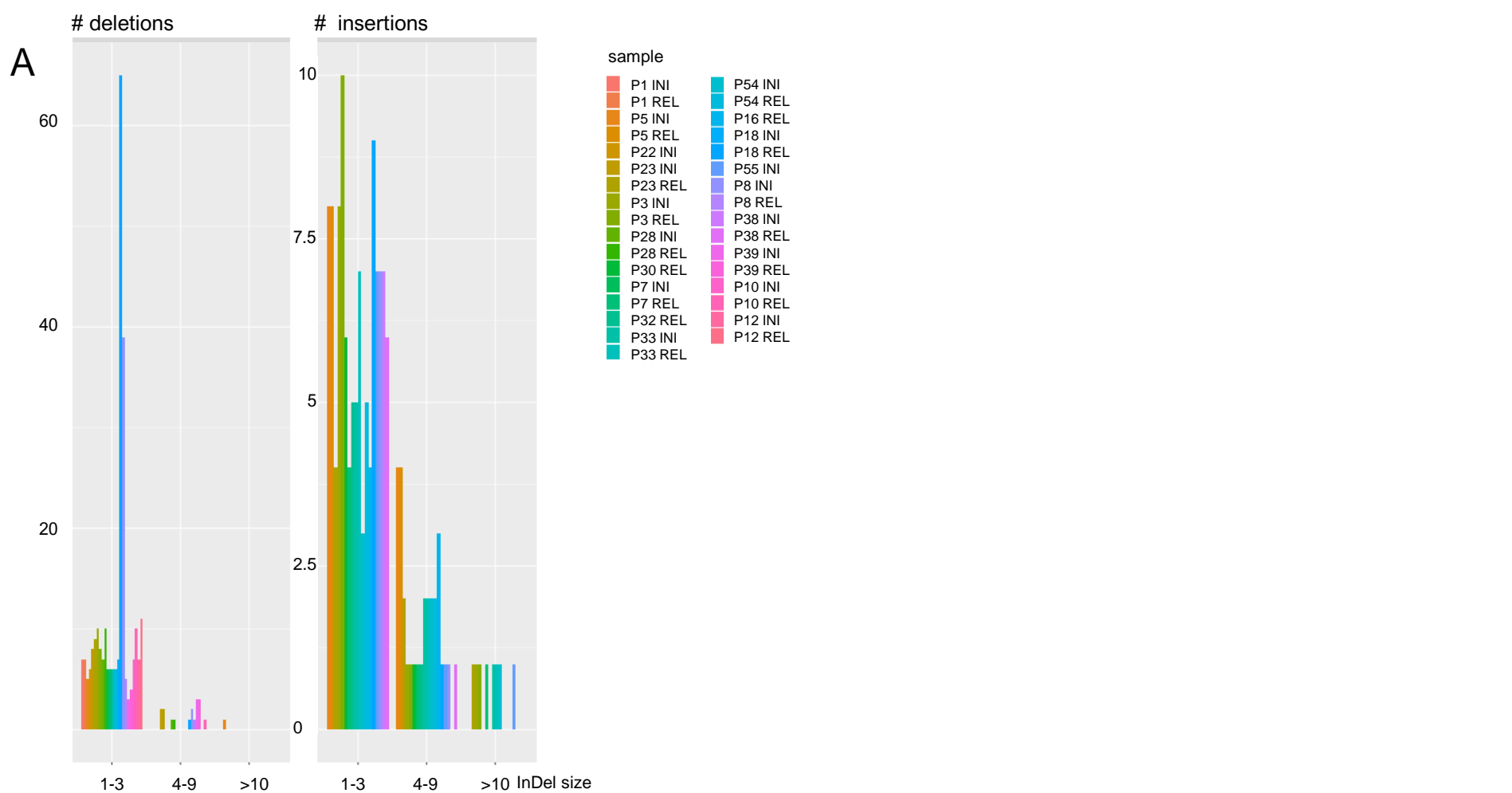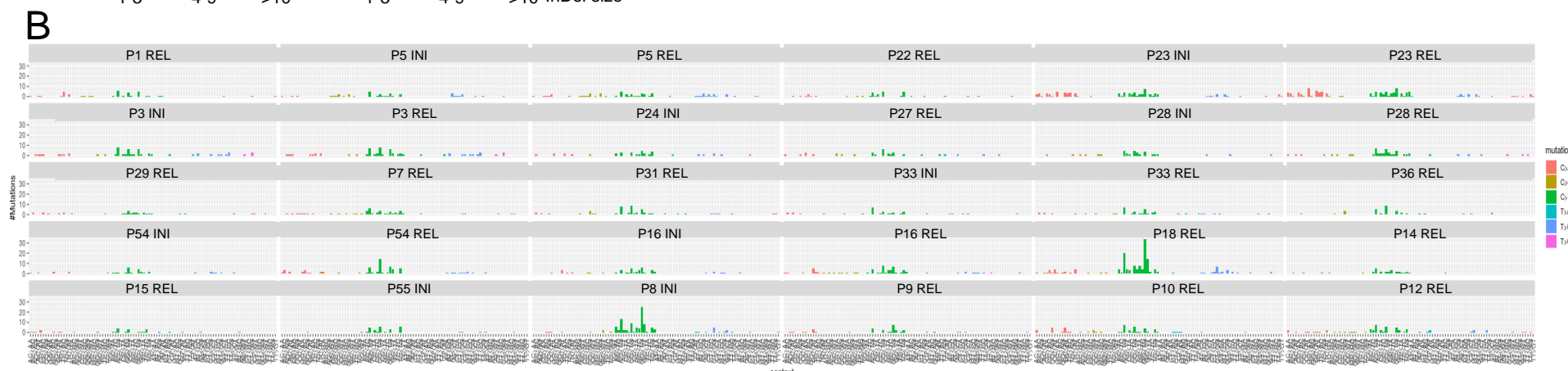

Supplement: Supplementary file 13 — Suppl. Fig. 2 [file 41375_2022_1587_MOESM13_ESM.pdf]

P27 initial diagnosis  
P27 relapse  
P27 remission

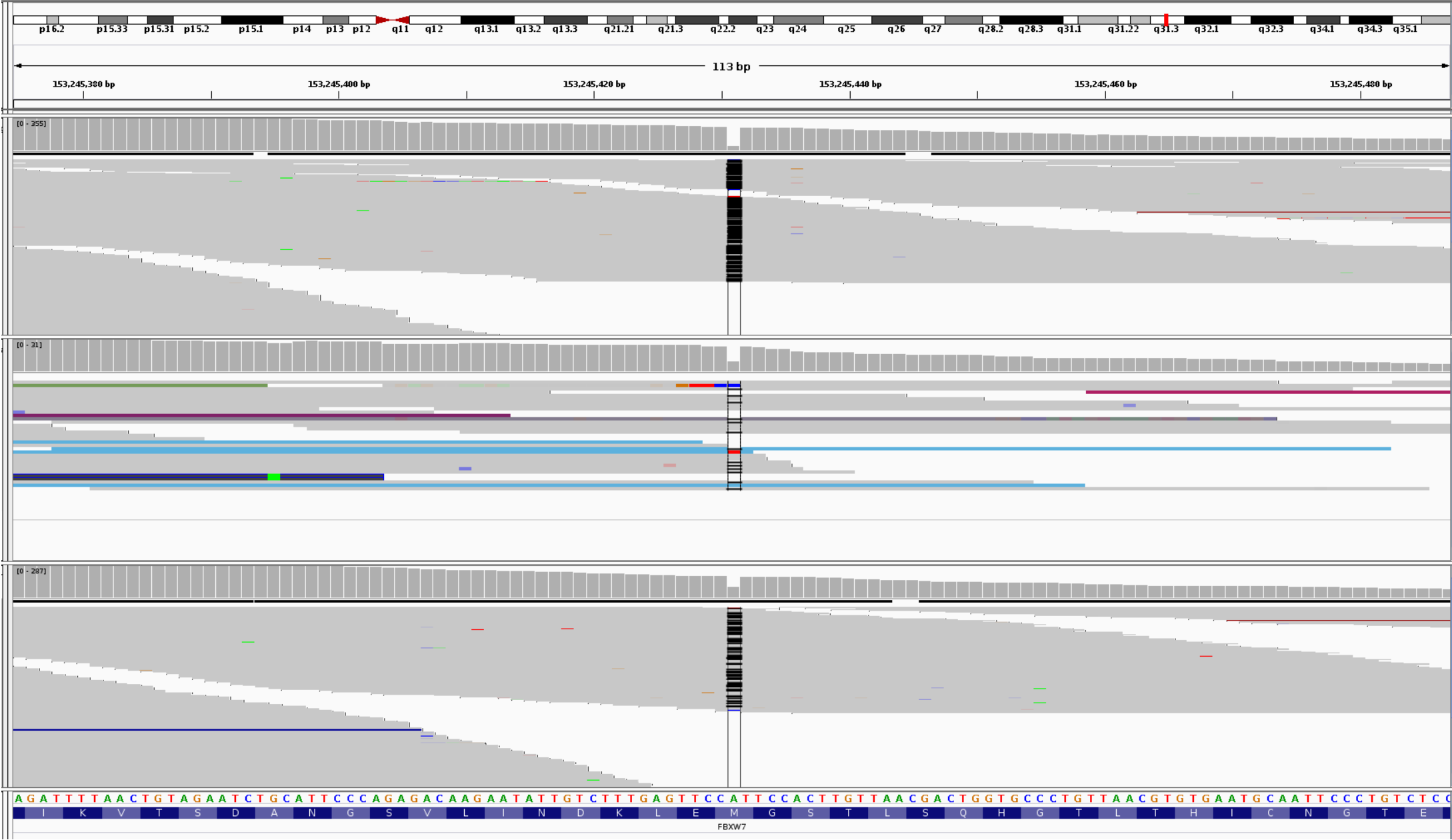

Supplement: Supplementary file 14 — Suppl. Fig. 3 [file 41375_2022_1587_MOESM14_ESM.pdf]

up-regulated in ETP-ALL

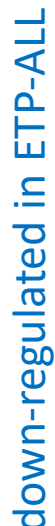

Supplement: Supplementary file 15 — Suppl. Fig. 4 [file 41375_2022_1587_MOESM15_ESM.pdf]

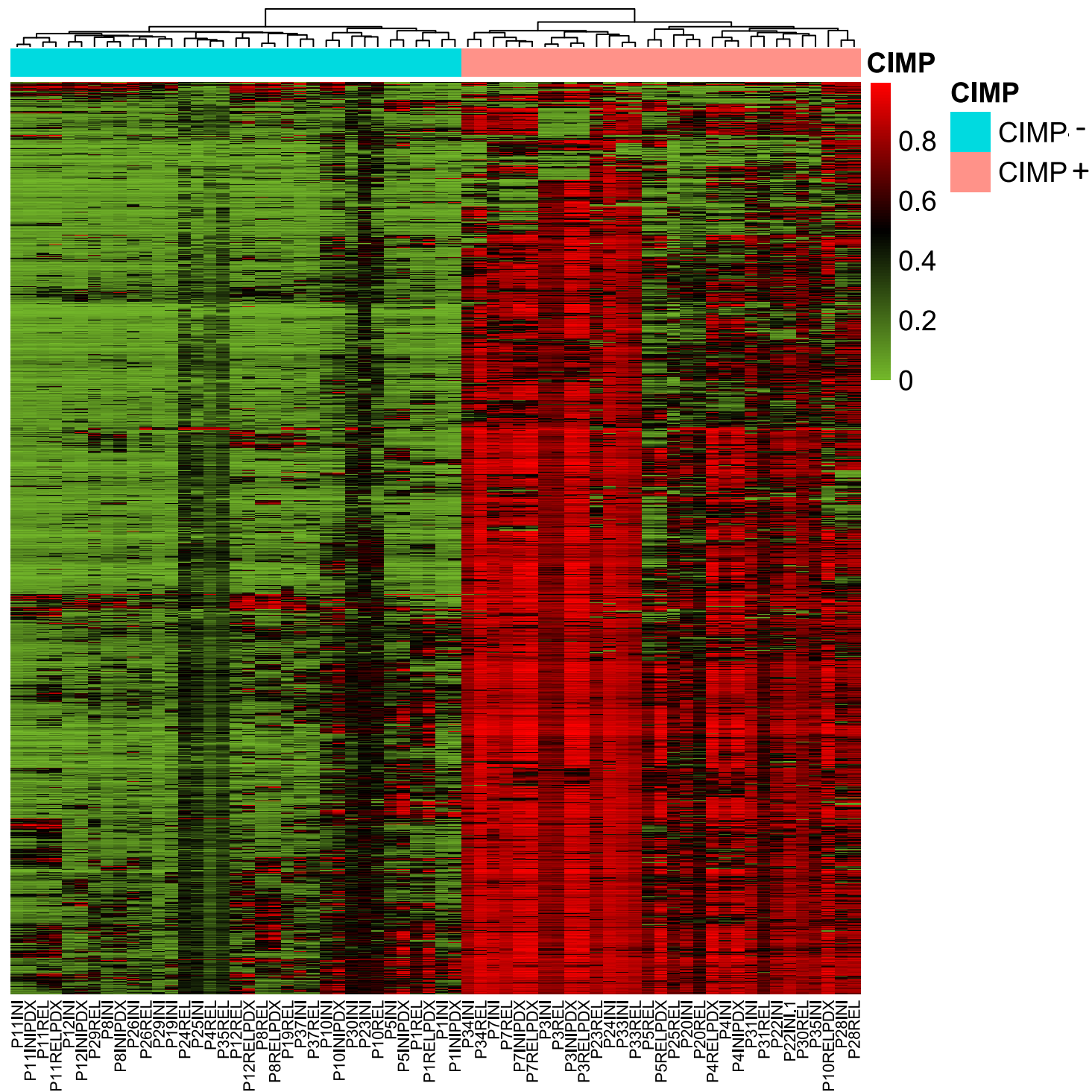

Supplement: Supplementary file 17 — Suppl. Fig. 6 [file 41375_2022_1587_MOESM17_ESM.pdf]

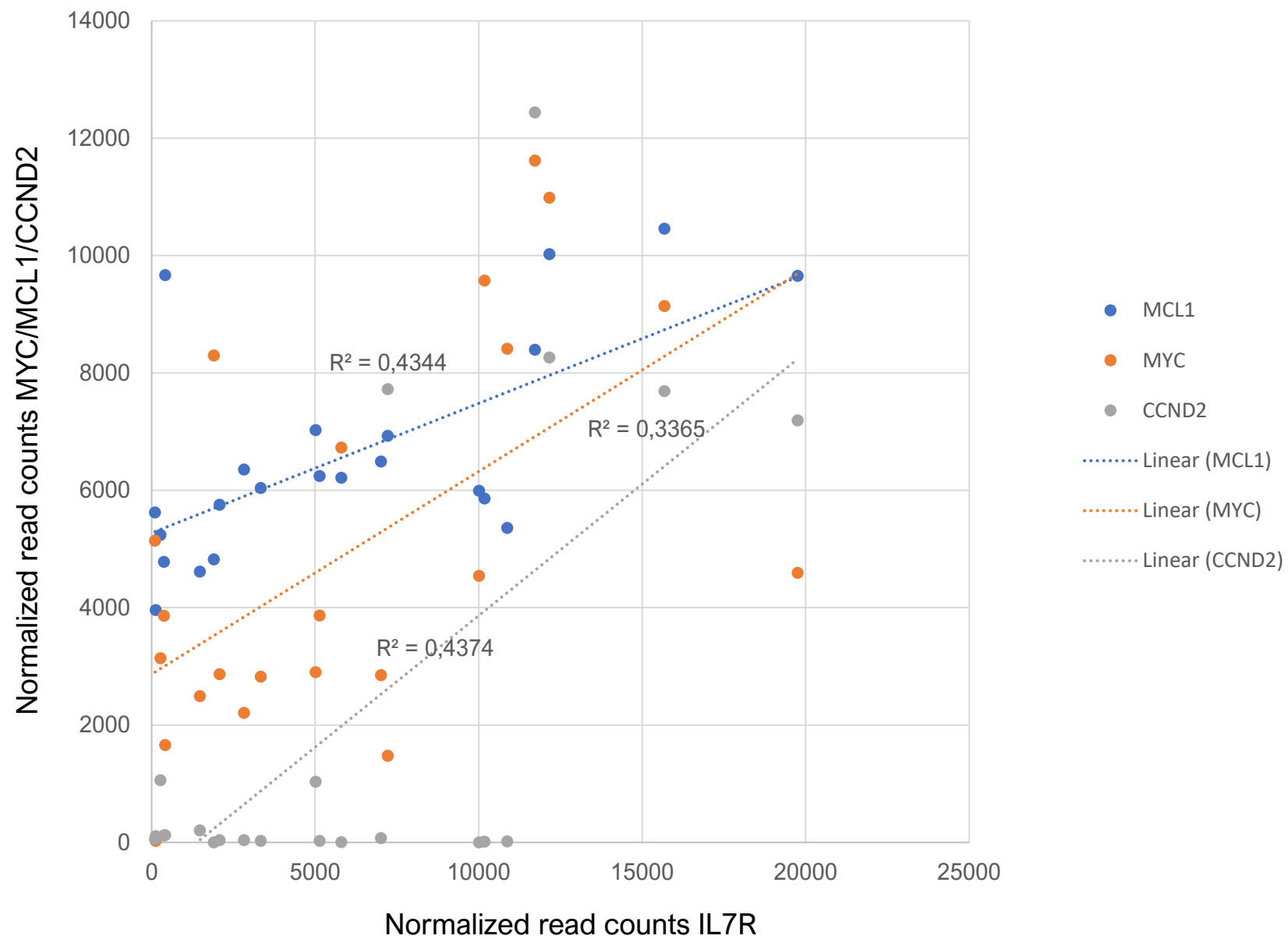

Supplement: Supplementary file 18 — Suppl. Fig. 7 [file 41375_2022_1587_MOESM18_ESM.pdf]
