# Supplementary material for: Pediatric T-ALL type-1 and type-2 relapses develop along distinct pathways of clonal evolution
Source: Leukemia. 2022 May 18;36(7):1759–68. doi: 10.1038/s41375-022-01587-0 (PMC9252914; doi:10.1038/s41375-022-01587-0)
Supplement: Supplementary file 16 — Suppl. Fig. 5 [file 41375_2022_1587_MOESM16_ESM.pdf]

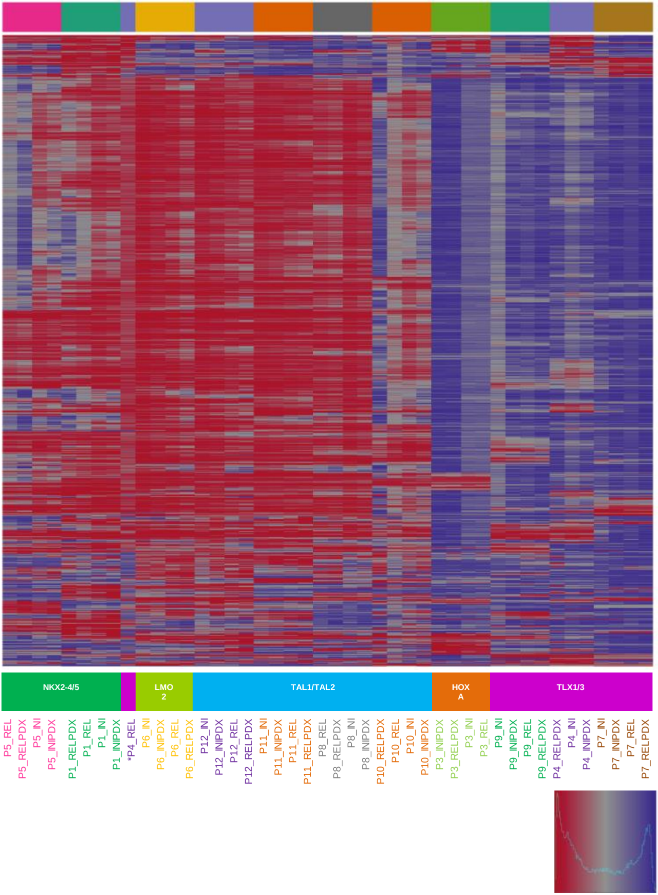
NKX2-4/5
LMO 2
TAL1/TAL2
HOX A
TLX1/3
P5_REL
P5_RELPDX
P5_INI
P5_INIPDX
P1_RELPDX
P1_REL
P1_INI
P1_INIPDX
*P4_REL
P6_INI
P6_INIPDX
P6_REL
P6_RELPDX
P12_INI
P12_INIPDX
P12_REL
P12_RELPDX
P11_INI
P11_INIPDX
P11_REL
P11_RELPDX
P8_REL
P8_RELPDX
P8_INI
P8_INIPDX
P10_RELPDX
P10_REL
P10_INI
P10_INIPDX
P3_INIPDX
P3_RELPDX
P3_INI
P3_REL
P9_INI
P9_INIPDX
P9_REL
P9_RELPDX
P4_RELPDX
P4_INI
P4_INIPDX
P7_INI
P7_INIPDX
P7_REL
P7_RELPDX
